# Abstracts and Gleanings

**Published:** 1878-12-20

**Authors:** 


### *ABSTRACTS AND GLEANINGS.*

---

### DERMALGIA, ETC., FROM QUININE.

BY HENRY M. FIELD, M.D.

*Professor of Therapeutics at Dartmouth.*

I have had within the year a case which well illustrates certain observations of Dr. Lente in the last number of the *Medical Record.* A young married lady, recently from Indiana, summoned me for a severe attack of "chills and fever." She told me almost at first she could not take quinine; that her father —giving the name of an Indianapolis physician, whom I knew by reputation —had despaired of using it in her case. She said, further, that it *poisoned* her; "it is as if every drop of blood and every tingling nerve were in my skin." There was great heat, œdema, in some places, especially in face and hands, great increase of curtaneous sensitiveness; all followed in a few days by desquamation of the cuticle. With these accidents were associated more or less of gastric disturbance, and, upon one occasion, violent and repeated vomiting, headache, and delirium.

Altogether, the suffering was so great, she said she preferred the "chills and fever." That her father had no resources for her except the imperfect and slowly acting arsenic and strychnia.

As my patient was intelligent, I related to her the course I had pursued in several similar previous cases, and proposed it for herself. It consisted in giving quinine in greatly lessened doses. She accepted my proposition, but evidently with fear and reluctance. Four to five grains sulphate quinine for the twenty-four hours acted with full therapeutic power, but still produced a light attack of the skin disease—enough to suggest alarming results should the usual one to three gramme doses be prescribed. She made a satisfactory recovery.

The course suggested is, I am satisfied, so far as one can be from a limited experience, the right course to pursue in these cases of idiosyncrasy.

The exceptional—even the abnormal—action of a remedy may be of valuable assistance to us in interpreting the laws and the phenomena of its usual physiological action. For that reason, such cases as that detailed may have an especial interest for us. We are not to suppose that the quinine, in this its extraordinary influence, is excreted from the skin, as some writers have taught. There is no sufficient evidence of this.

On the other hand, I believe—as I have tried to show in my monograph on certain salts of quinine about to be published by the Vermont Medical Society—that the main action of the great remedy is exerted upon the sympathetic, and not upon the cerebro-spinal system of nerves. I think no one can attentively study Briquet's treaties without coming to the conclusion that such was his opinion, though it be not anywhere expressed in words. I cannot do more here than express this fact of my belief—although it involves many important considerations—that the dermalgia, urticaria, œdema, etc., which sometimes result from what should not only act as a medicinal dose, serve to illustrate and enforce it.

Then, again, quinine makes a signal impression upon the vaso-motor system, as well of the cutaneous surface as of the nerve-centres—an impression which involves facts of great importance both in physiology and therapeutics. It has its own appropriate influence upon the heart, the arteries, and the capillaries and arterioles. With this in view, it is not strange that a departure from its normal working should bring discomfort or disaster to the skin.

Then, finally, quinine has a special action upon the skin as truly as arsenic has, but not of such significance of course, and thus far insufficiently studied. I am satisfied certain remedial resources await investigation in this direction. The praise which Mr. Erastus Wilson accords to quinia for its power to cure that neurosis of the skin known as *prurieus,* and Mr. Spender's success with it in *pruritus ani* will serve to illustrate this statement.—*N. Y. Med. Record.*

---

### COMPARISON OF OPIUM, BELLADONNA AND ACONITE.

Mr. Jules Simon establishes the following comparison between opium, belladonna and aconite:

1. In regard to their action on the alimentary canal, opium causes thirst, dryness without acrimony, want of appetite, nausea, vomiting, constipation; belladonna, thirst, dryness with acrimony, nausea, vomiting, and diarrhœa; aconite, dryness, sensations of pricking, and burning of the tongue, salivation in full doses, vomiting, and diarrhœa.

2. In regard to their action on the circulation, opium acts as a stimulant, causes diminuition of pressure, though it is sometimes without action, and in large doses causes acceleration of the pulse and collapse. Belladonna acts as a sedative, lowers the strength, retards the frequency of the pulse, and produces a febrile state of the system. Aconite acts as a sedative, diminishes the arterial tension, renders the face pallid, retards the frequency of the pulse, and stops the heart in diastole.

3. In regard to the respiration, opium allays dyspnœa, when present, by diminishing the bronchial secretions; in large doses in causes collapse. Belladonna calms down excited respiration, diminishes secretion, and in large doses renders respiration spasmodic and irregular. Aconite retards respiration by its direct action on the nerves.

4. In regard to their action in febrile states, opium augments the cutaneous secretions, and produces general malaise, erythema, and eruptions. Belladonna produces neither sweating nor general discomfort, raises the temperature, and sometimes causes scarlatina-like eruptions. Aconite lowers the temperature.

5. In regard to their actions on the secretions, opium diminishes the quantity of urine, and, in fact, diminishes the secretions generally. Belladonna causes augmentation of the renal secretion, with diminution of the bronchial secretion. Aconite causes increase of the urinary secretion, but diminishes the bronchial secretion.

6. In regard to their action on the nervous system, opium acts chiefly on the cerebro-spinal system, belladonna on the cerebro-spinal system, and aconite on the spinal cord. Opium causes somnolence, sleep, intoxication, vertigo, muscular debility, diminution of common sensibility, contraction of the pupil, diminution of the activity and vigor of reflex actions. Belladonna causes sleeplessness, gay or furious delirium, hallucinations, muscular agitation, diminution of the sensibility of the face, diluted pupils, and remarkable diminution of the reflex acts. Aconite leaves the intellectual faculties intact, but causes muscular torpor, anæsthesis, hullucination of the senses, diminution of reflex actions, and produces slight dilatation of the pupil. *London Lancet.*

---

### IODOFORM FOR SYPHLITIC SORES.

---

Mr. Berkely Hill has used iodoform as a dry powder, brushed lightly over the suyface with a moistened camel-hair pencil, for three years. During the last few months he has often substituted for the dry powder an etheral solution, one part of iodoform in six or eight of ether. The sore is touched or dabbed with a pencil dipped in the ethereal solution, according to its size and depth, lightly or copiously. The ether quickly evaporates, leaving a thin pellicle of iodoform, that as effectually stays the spread, and produces healing of chancres, as does the more copiously applied dry powder. Thus the surface is covered more exactly, and the disagreeable smell of the iodoform is too faint to attract attention. The sore is well washed with water and dried before the iodoform is applied, and the surface is lastly protected by a bit of dry lint. When the secretion is abundant, the dressing must be renewed twice daily, but in three or four days the amount of discharge becomes so scant that one dressing per diem suffices. In this way Mr. Hill finds venereal sores heal quickly. Pain subsides at once; the sore is well in a week or ten days, and the chances of consecutive inoculation or bubo greatly lessened. (The Doctor.)—*Practitioner.*

---

Treatment of Tinea Favosa.—Dr. Viger reports two cases of tinea favosa that were cured by external applications of the oil de cade. In both cases almost the entire head was covered with thick coats of favus. The local treatment was as follows: The head was washed morning and evening with soap and water, after which it was ointed with oil of cade, and then covered with a cap of oiled silk in a such a manner as to prevent the admission of air.—Internally, cod-liver oil and a nourishing diet were prescibed. Under this treatment the crusts rapidly disappeared, and both the patients were discharged cured, after two months treatment. One of these cases was treated eighteen months ago, and the other six months ago, and the other six months ago, and neither of them have as yet shown any symptoms of a relapse.—*L'annee Med.*, April.

### ON THE USE OF CALCIUM CHLORIDE IN THE TREATMENT OF PHTHISIS, TABES MESENTERICA, CHRONIC DIARRHŒA, ETC.

Dr. Mellersh says: Attention has been called to the use of calcium chloride in certain forms of disease, but especially to its use in tabes mesenterica. The remedy was formerly much used, but had fallen into disuse. As it is now again on trial in this country and in Europe, perhaps the notes of a few cases in which it has been used may not be uninteresting, especially as these cases seem to warrant an extended trial of its virtues.

E. M., a girl of thirteen years, a strumous subject, has been suffering for two years with chronic diarrhœa and tumid abdomen; both lungs are tuberculous, the apices being largely involved; she was reduced to almost the last degree of emaciation. Has been under the usual treatment of cod-liver oil, tincture of bark, syrup of the iodide of iron, with milk diet, etc. She was not able to leave the bed.

After taking the calcium chloride the diarrhœa stopped, and there was a decided improvement, so much so that in the course of two weeks she was able to walk to the dispensary, a distance of six blocks.

In a number of phthisis pulmonalis cases, both in and out of the hospital, the remedy has appeared to act beneficially, and in several cases the patients have asked for the remedy after it had been discontinued.

In a case of typhoid fever, under Dr. J. S. Cohen's charge, after other remedies had failed the calcium chloride relieved the diarrhœa at once.

In some of the chronic and acute diarrhœas of children it has appeared to me to be very salutary in its effects, and I shall be glad to hear the results of more extended trials in this direction.

The cheapness and innoxious character of the drug also recommend it, especially in dispensary practice; the solution is the best given in thirty-drop doses, in milk, for an adult. For children it may substitute the stereotyped aqua calcis and milk, or it may be given in emulsion with cod-liver oil.—*Medical and Snrgical Reporter.*

### TUBERCULAR ULCER OF THE TONGUE.

M. Nedopil, in the *Archiv. for Klinische Chirurgie,* remarks that the diagnosis of secondary tubucular ulcer of the tongue is generally not difficult in the presence of other indications of tuberculosis. On the other hand, primary tubercular ulcer can often be scarcely distinguished from cancer unless a microscopic examination be made; while the failure of anti-syphilitic treatment, distinguises it from syphilitic ulcer, which often has a similar appearance. The tubercular ulcer of the tongue runs a course resembling that of cancer. A small hard nodule on the edge or upper surface of the tongue, which is often overlooked, at last falls off, and leaves a dirty ulcer, with an indurated base, which generally spreads more slowly than a cancerous ucer. A cure can be produced only by early extirpation, which, perhaps, may arrest the developement of general tuberculosis. The author has observed four cases in Bilroth's clinic; two of the individuals were thirty-two years of age, the others sixty eight and seventy. In three cases the ulcer was extirpated, and healing took place in a few days. In the excised pieces the tissue around the ulcer was studded with miliary tubercles, mostly toward the free surface. The morbid process appears to commence with a general transformation of the muscular tissue into a homogenous slightly granular deposit containing preliferating muscle-nuclei. Later, the primary deposits become confluent, and giant cells are formed from the obstructed portions of the blood-vessels; in some of these Nedodil found cavities filled with brown pigment. The growth of the tubercle appears to take place partly through proliferation of nuclei (without cell formation) in the interior, partly through metamorphosis of the neighboring tissue.—*The Doctor.*

---

Tartarized Antimony.—Tartarized antimony is a valuable agent which has fallen greatly into disuse of late years. Administered in small doses its action is most marked and beneficent. Apart from its catalytic effect in the blood, it acts as a sedative, diaphoretic and expectorant. To produce its true power, it must, according to Læennec, be absorbed into the blood, and this can only be brought about by the administration of minimum doses. Its efficacy in small doses, as an adjunct to purgative remedies, through its power of relaxing the muscular fibre of the intestines, is very generally known. At one time tartarized antimony entered into the composition of every febrifuge and expectorant mixture, but owing to its being prescribed in too large portions, and consequently producing great vital depression, it lost character as a medical agent. This loss of character was, perhaps, also partly due to the great revulsion that has taken place in the practice of medicine in the last quarter of a century. The abandonment of tartarized antimony we conceive to be a very great error. We prescribe it daily, in very small doses, with the happiest results, and believe, when properly given, it possesses special merits. In disregarding its use the profession, in our judgment, has lost one of its most useful remedies.—*Trans. A. M. Ass.*

---

Removal of Moles.—The acid nitrate of mercury is recommended as effectual for the removal of moles. It should be applied with a splinter of wood in small quantity for a few seconds, carefully avoiding the sound skin. There is no pain of consequence, and the mole shrivels away and drops off in a few days.

### TREATMENT OF NÆVUS BY SODIUM ETHYLATE.

Sodium ethylate, which was first obtained and used by Dr. Brunton in 1871, is prepared by adding the metal sodium, piece by peice, to some absolute alcohol in a wide-mouthed bottle; cautious addition of more sodium until effervescence ceases results in the deposition of a crystalline substance—$C_2H_5NaO$—at the bottom of the flask.

The credit of bringing this substance and other alcoholic and ethylic derivatives before notice was due to Dr. Richardson, who, in a communication on the subject to Dr. Brunton, writes: "When it is brought into contact with water it is decomposed, the sodium becoming oxidised by the oxygen of the water to form sodium hydrate, and the hydrogen of the water going to reconstitute the common or ethylic alcohol. The change of ethylic alcohol into sodium alcohol transforms it from an irritant to a caustic. Laid on dry parts of the body, the sodium ethylate is comparatively inert, creating no more change than the redness and tingling caused by common alcohol; but as soon as the part to which the substance is applied gives up a little water, the transformation described above occurs, caustic soda is produced in contact with the skin in proportion as water is eliminated, and there proceeds a gradual destruction of tissues, which may be moderated so as hardly to be perceptible, or may be so intensified as to act almost like a cutting instrument." Speaking of the practical uses of sodium and potassium alcohols, the same writer says that he does not yet see the means of applying them internally, but predicts for them a very extended application for external purposes, they being most potent caustics, *e. g.* for the destruction and removal of malignant growths beyond the reach of the knife by application to the surface, or by subcutaneous injection into the growths. Applied direct to the unbroken skin, their destructive action is less painful than would be expected, and when pain is felt it may be checked quickly by dropping on the part a little chloroform, which decomposes the alcohol, converting it into a chloride salt and ether.—*Louisville Med. News.*

### SPINAL CURVATURES.

Dr. Macleod tried other substances besides plaster of Paris, such as paraffin, glue, starch. Glue did pretty well, but was not equal to plaster; while paraffin did not do well, and was dirty to handle. He also pointed out that insteaa of Sayre's suspension apparatus, it was easy to improvise with a room door an arrangement that would serve the purpose. As regarded abcesses, which sometimes occurred in Pott's disease, Sayre, who did not believe in antiseptic surgery, opened them freely, and cleansed out the abcess with Peruvian balsam (an antiseptic). Dr. Macleod then demonstrated minutely the further treatment for abscesses. He also showed by means of a model that, as proved by Sayre, in what was usually called lateral curvature, there was a rotation of the bodies of the vertebræ upon themselves. On this account he (Sayre) had substituted the term "Rotary Lateral" for lateral, as being discriptive of the exact state of matters. In regard to this kind of curvature, all the ordinary kinds of apparatus went on the wrong principle, and did harm. The object was to get back muscular tone, and this was done by exercising the muscles which had lost their energy. Mere lateral pressure would do no good at all. The spine must be straightened by self-suspension several times daily, for months at a time. The hand on the concave side should be held uppermost. After a considerable experience of these cases of curvature, he had no hesitation in saying that Sayre's treatment of them was very far in advance of any former methods of treatment which he had tried.—*Glasgow Medical Journal.*

Epithelioma.—Dr. Duhring, in the Pennsylvania Hospital, remarked:

Epithelioma, as a rule, was found on older persons. The affection belonged to the flat or superficial variety of epithelioma, and the treatment appropriate to the case was cauterization by means of caustic potassa. The knife was not called for in this instance. The solid stick of potassa fusa was then applied, going slowly over every portion of the diseased structure, and allowing the influence of the caustic to penetrate a little below and beyond the diseased tissue in every direction. It was not necessary, Dr. D. said, to use any pressure or force, but the caustic should be brought most carefully in contact with every particle of the growth, lest recurrence should take place, and a second operation be required. When the action of the caustic had gone far enough, it was limited and checked by the application of dilute acetic acid. The pain of the operation was quite sharp and severe, but ceased almost immediately upon the application of the acetic acid. The patient was directed to dress the part simply with lint soaked in olive oil; the dressing to be removed twice daily and the wound cleansed.—*Med. and Surg. Reporter.*

---

The Ointment of Nitrate of Silver is a new thing in pharmacy. When nitrate of silver is attempted to be mixed with lard or most organic bodies it is decomposed, and the ointment turns black in color. The property of vaseline not being decomposed by nitrate of silver was discovered by a French physician, who desired to use it in ophthalmic practice. He found it inalterable, and wrote to Mr. E. Fougera announcing his discovery. I have succeeded in making an ointment containing one part of the powdered crystalized nitrate of silver to two parts of vaseline. Suppositories of nitrate of silver for urethral and uterine diseases can be made with paraffine and vaseline as a base.—*Drug Cir.*

Intestinal Gas—Flatulent Dyspepsia.—Dr. Eads (in Sc. Med. Jour.) in a case of the above character "prescribed tinct. Colocynth gtt. x., water ℥iv.; gave a teaspoonful every three or four hours until relieved. Next day found my patient perfectly free from pain, no wind in stomach or bowels—the first permanent relief obtained for two months. The treatment was continued for a few days only, the patient thinking herself well, discontinued it. In a short time the flatulency returned, but not as severe as before. Ordered the above prescription to be taken whenever there is any gas in the stomach or bowels. Pregnancy was now in the eighth month. Hoping there would be no further trouble after confinement, that time was looked for with great interest.

January 10th, 1878, delivered patient of a fine 8-lb. male child; recovery after confinement good, even better than was expected; considerable trouble the first three weeks with intestinal gas, but relieved every time with colocynth. When the babe was about three weeks old it began to suffer very mnch like the mother with an accumulation of intestinal gas. Colocynth gtt. ij, water ℥ij., a teaspoonful three times a day, gave prompt relief. Now nine months since, mother and child both well. I believe colocynth to be a specific in the above condition."

---

Ipecac.—The effects of ipecac on the economy are very similar to those of antimony, save that it does not act on the blood, and therefore can not be ranked as an antiphlogistic. As a neurotic, diaphoretic and expectorant, its power is unmistakable. Its influence on the mucous membrane, in minute doses, is most interesting. We have seen cases of persistent vomiting, which had continued for days, arrested by small quantities of this drug, all other means having failed. Very large doses of ipecac have been suggested recently in dysentery, and its use in this way, if we are to believe the journals, has been very successful. We have had no personal experience in this matter, but we can readily understand in what manner nauseating or emetic doses of ipecacuanha might, by their relaxing effect on the whole system, influence the rectum itself. Small quantities of ipecac, in combination with opium, have almost a specific action in dysentery, particularly if they follow the administration of a saline aperient. In no form is the potency of small doses of ipecac so apparent as in the troche or lozenge, where its specific action on the mucous surface of the throat and bronchi, becomes at once evident.—*Ib.*

---

Ovariotomy Superseded.—A proposal has been brought before the Paris Academy of Sciences by M. Tripier to establish a fistular between the cavity of an ovarian sac and the exterior. He has tried it in one case with success. The interior of the sac can in this way be washed out or treated with iodine injections or cauterised. He has used injections of iodised water daily. The galvano-caustic is used to establish the fistula. This operation is less formidable than ovariotomy, and can be easily carried out, but, of course, is not devoid of danger, but it may be applicable in cases where gastrotomy is refused or inapplicable. With regard to injections, they should not be too strong. We may point out that death from poisoning by iodine has been recorded where the drug was injected. This operation may be compared with electrolysis for ovarian dropsy.—*The Doctor.*

---

Urea Formation.—Prof. Gamgee, of Owen's College Manchester, has recently published an account of some new experiments to determine the seat of the urea formation in the body. The result is, the experiments have demonstrated that the liver is the principal if not the only organ of the body concerned in urea formation.—*Lancet and Clinic.*

The Treatment of Diarrhœa by Oxide of Zinc.—Dr. Jacquier has followed, in the service of Dr. Bonamy at Nantes, the good effect of the employment of oxide of zinc in diarrhœa. The fomula which he has employed is the following: Oxide of zinc, 10 grains; bicarbonate of soda, 7½ grains; in four packets, one to be taken every six hours. In all the cases which he observed, oxide of zinc produced rapid cure of diarrhœa. In fourteen cases observed by Pupgautier, the cure was even more rapid, since in only one case were three doses of the medicine required. The results are considered to have been more satisfactory, inasmuch as in several cases the malady had endured from one to many months, and other methods of treatment had not produced any improvement. Thus he concludes that, although by no means to be held as exclusive treatment, the employment of oxide of zinc deserves to be more generally known as useful in diarrhœa.—*British Med. Journal*, Sept. 28, 1878.

---

Fibroid Tumors of the Uterus Treated by Sclerotic Acid.—Dr. John Williams reports two cases of uterine fibroid treated by sclerotic acid. This acid dissolves readily in water, and in so far differs from ergotine. One of the cases was that of a woman aged 34, who had suffered from severe flooding for some time. A fibroid tumor was detected, and half-grain doses of the acid were injected under the skin of the abdomen twice a week. The flooding was much reduced, but returned during a temporary discontinuance of the treatment. When the injections were again commenced, the flooding was checked as before. The tumor was reduced in size. Like results were attained in a similar case, including a decrease in the size of the tumor. The injection caused a little pain at the time, but that was all. It was followed in about half an hour by uterine contractions.—*The British Med. Jour.*, May 18th.

Nursing Sore Mouth as seen in patients who seem well in other respects; is generally an index of lowered vitality—sometimes in inflamation, the result of local nutritive disturbances of reflex origin; the irritation being mammary. In the ptyalism of pregnacy we see an instance of the same etiological kind. S'omatitis materna takes the form of acute or chronic aural catarrh, also aphthous and ulcerative stomatitis. It is sometimes very obstinate and intractable. Weaning the child always brings about a speedy cure. It is generally, however, amenable to treatment of a simple nature. Where, from lowered vitality, a tonic regimen—as quinine, porter, iron and cod-liver oil—is required, there are two preparations of the latter in the market which I find almost equally serviaeable—Scott's Emulsion and Phillips's. Many, however, can take Phillips's oil that cannot take Scott's. Phillips's oil is borne by the most delicate stomachs. I consider this preparation invaluable for nursing women whose nutrition is below par.—*N. Y. Med. Rec.*

---

Death from Chlororm. — T. Hughes, M. D., in London Lancet of November 2d, says: If I were about to be placed under the influence of chloroform, I would say, "Never mind my pulse, never mind my heart; leave my pupil to itself. Keep your eye on my breathii.g; and if it becomes embarrassed to a grave extent, take an artery forceps and pull my tongue well out." It was the observance of this simple yet all-important rule that enabled the late Mr. Syne to say that he never lost a single case from chloroform, although he gave it in five thousand cases. Prof. Lister has done much to enforce this rule of practice, and to him is due the credit of pointing out the *modus operandi* of this proceeding. He was the first, as I am aware, who explained that its action is not mechanical, but is exerted chiefly through the nervous system.—*Louisville Med. News.*

Treatment of Pityriasis Versicolor.—Prof. Hardy treats this affection with sulphur-baths, or frictions, several times a day, with the following ointment, ℞. Axung. ℥ i., sulphur, ʒ ss.; acidi nitrici, gtt. x. m. Internally he gives mineral waters if the case be a severe one, and follows them up with Fowler's or Pearson's solution of arsenic, in doses progressively increasing from three to ten drops. The drops are taken before each meal in a glass of wine and water.

Dr. Besnier, of the Hopital Saint Louis, Paris, gives the preference to sublimate baths, or, where these cannot be used, to frictions with a soap containing pumice-stone, followed by lotions with a solution of corrosive sublimate, gr. iv. to ℥ iv. distilled water. Where it is not considered safe to leave so poisonous a mixture in the hands of the patient, Dr. Besnier substitutes the following simple treatment: Lotions with soft soap, followed by three applications of an ointment containing ʒss. of Turpeth mineral to the ounce of lard. In one of his patients, whose entire chest and back were covered with the patches of pityriasis, a cure was produced by two lotions and only two frictions.—*Lyon Med.*, May 5th.

---

Acetic Acid Injections in Carcinoma.—Acetic acid, used subcutaneously, in cancer, is by no means a novelty; but does not deserve to be forgotten. It has been revived of late by Dr. T. Gies, who states, in the *Zietschrift fur Chirurgie*, that he effected marked improvement in a case of cancer seated on the ramus of the lower jaw, which had returned after operation, and had attained the size of a fowl's egg, by the injection of acetic acid in the proportion of one part of the acid to three of water. The tumor speedily underwent reduction of size, and was ultimately no larger than a hazel nut. The same patient had a second tumor, of the size of an egg, beneath the ear, which almost entirely disappeared in the course of twenty-one days, after twenty-five injections of strong acetic acid. In a woman, again, who had a carcinoma of the size of a pigeon's egg, in the left mammary gland, ten injections effected its removal, in great part, in the course of a month, reducing it to the size of a hazel nut.

---

Treatment of Intertrigo in Children.—Dr. Wertheimber recommends the external use of a solution ot corrosive sublimate (gr. i. to ℥ iijss. water) for the intertrigo of children, and asserts that it is the most effective of all the remedies recommended for that affection. The affected spots are covered with peices of lint soaked in the solution. Sometimes it is sufficient to keep these compresses on for an hour at a time, three our four times a day. The curative action is very rapid, the redness and exudation often disappearing in 24 to 36 hours. General symptoms, due to the absorbtion of the drug, have never been observed by Dr. Wertheimber; the danger of absorption is not great, as the applications do not, as a rule, need to be continued more than a few days. When the improvement is pretty well advanced, dyachylon ointment may be substituted for the solution.—*Berliner klin. Wochenschrift, No.* 15.

---

Sulphurous Acid for Abscesses.—At a recent meeting of the Clinical Society of London, Mr. Osman Vincent described a method by which he had opened eighteen lumbar abscesses without a fatal result. The abscess was first opened and then injected with a solution of equal parts of sulphurous acid and water, after which a poultice was put on. Next day the injection was renewed and some tenax applied. The treatment went on till the cavity healed up. The injection sometimes gave pain. Sometimes the fluid returned clear, and at other times black. When sulphurous acid was injected, it acted upon the pyogenic membrane, and then pus did not reform.

TREATMENT OF SORE NIPPLES.---Dr. Haussman, of Berlin, recommends very highly the use of lotions containing five per cent, of carbolic acid, in the treatment of erosions of the nipples. He claims that the carbolic acid not only cauterizes superficially the eroded spot, but that it penetrates into the openings of the smallest lymph-vesels which have been laid open by the erosion, and destroys at once any parasitic germs or infectious organic substances that have been conveyed to the nipple by the mouth of the child or the hands of the physicians or nurse, or of the woman herself. In so doing it prevents the developement of almost all inflamations of the mamary gland itself. Of course the nipple must be carefully cleansed every time the child is put to the breast.—*Centralblatt for Gynak., No.* 10.

---

PYROGALLIC ACID IN PSORIASIS.—New Remedies: We have already in our July number, upon page 208, given a *resume* of some experiments made with pyrogallic acid as a substitute for chrysophanic acid in psoriasis. The author, Dr. A. Jarisch, now reports his complete success in the treatment of this affection by the agent indicated. At first he used an ointment containing twenty per cent of pyrogallic acid. This was, howover, found to produce excoriations. Hence he has reduced the ointment as ordinarily used to the strength of ten per cent. If spread on muslin, and then applied, it must be still further diluted; otherwise it acts as an irritant. Aqueous solutions should contain about one per cent. Pyrogallic acts not as rapidly as chrysophanic acid, but it is equally certain in its results.—*Louisville Med. News.*

---

LIQUOR SANTAL FLAVA CUM BUCHU ET CUBEBA.—This preparation appears likely to become a favorite prescription in cases of gonorrhœa and gleet. It contains three remedies of proved utility in these diseases, the santal oil especially having a very extraordinary power to arrest certain cases of gleet. Experience has shown this preparation to possess the same efficacy as the santal oil itself. It mixes perfectly with water and has a taste by no means disagreeable, in which particular it contrasts very favorably with the ordinary mixtures it is intended to replace.—*Lancet and Clinic.*

---

CODEIA FOR CANCER OF STOMACH.—Dr. Austin Flint says: With reference to treatment, it is merely palliative. Pain in this instance is a symptom to be palliated; so also is the vomiting. I should say prescribing for the patient as now presented, that some form of opium is indicated. I prefer to use codeia, for the reason that it is much less liable to produce disagreeable after-effects than are some of the other preparations of opium. I should recommend, therefore, that this man take $\frac{1}{4}$ of a grain of codeia twice a day.

---

EVIDENCES are accumulating in favor of nitrite of amyl as a prompt and valuable cardiac stimulant in cases where a quick action is needed. Unfortunately, however, too many physicians are afraid of this useful preparation, notwithstanding it has never done any harm, even when inhaled in half-drachm and drachm doses.—*Louisville Med. News.*

---

CHLOROFORM NARCOSIS. — N. Y. Medical Record: Wachsmuth, of Berlin, asserts that much of the danger from the administration of chloroform may be averted by adding to it twenty per cent of oil of turpentine, which, he says, stimulates the lungs, and thus protects them against the great enemy of chloroform narcosis—pulmonary paralysis.—*Louisville Med. News*

---

Dr. S. Ringer says, "in ulceration and sloughing of the cornea, lead washes must be avoided, lest a white compound become deposited in the structures of the ulcer, leaving a permanent opacity."

### PANCREATIC FLUID.

The pancreatic liquid, according to Th. Defresne, contains three distinct ferments, of which *myopsin* dissolves albumen *amylopsin* saccharifies starch, and *steapsin* decomposes fats.

*Myopsin* forms garnet-colored shining scales, which are soluble in water, the solution being coagulated by heat; it digests 104 times its weight of albumen, but does not affect starch or fat.

*Steapsin*, when dry, is in translucent shining scales, which are soluble in water. It has no action on starch, but decomposes 24 times its weight of fat. It is precipitated and rendered inactive by acetic acid.

After washing and drying, *amylopsin* forms lemon-yellow shining scales, which saccharify 25 times their own weight of starch, are soluble in water, the solution being precipitated by alcohol and strong acetic acid, and coagulated by heat.—*Rep. de Phar.*, June, 244-246.

---

OVARIAN DYSPEPSIA.—Dr. Milner Fothergill describes a form of dyspepsia combined with leucorrhœa, and commonly too with menorrhagia, which depended upon morbid conditions of one or both ovaries. Experiment has shown that irritation of the sympathetic nerves of the stomach produces contraction of the gastic arterioles and defective secretion of grastic juice. In aggravated cases, there was vomiting of a reflex character, as seen in the early months of pregnancy and in calculus of the kidney. This form of dyspepsia was very intractable, unless its casual relationship were remembered and borne in mind in the treatment. Blisters over the ovary with bromide of potassium, and sulphate of magnesia internally, were more effective than bismuth and hydrocyanic acid.—*Med. Ex.* (*London*), Nov. 1, 1878.

---

Iodoform is given in doses of from 5 to 10 centigrammes ($\frac{4}{5}$ to $1\frac{1}{2}$ grain) three or four times daily, in solution of ether, in powder, or in pills. For ointment, one part of iodoform is mixed with eight or ten parts of fat at the temperature of a water bath. Rubbed to a fine powder, it is used for sprinkling and dressing varicose ulcers, cancerous and syphilitic ulcers, anal fissure, etc. Mixed with lycopodium, it is used for insufflation in vagina, and for sprinkling in the vulvitis of children.

---

BI. CARB. OF SODA.—In acid or flatutent dyspepsia, nothing has been found so useful, at least as a palliative, as a half or one teaspoonful of the bi. carb. of soda. For heartburn it gives immediate relief. The opinion of some that it injures the coats of the stomach is a mistake. The writer knows a man who for twenty years has been taking it daily for acid dyspepsia. Of late he has been in jail. The confinement seemed to increase the acid condition of his stomach; so that he is taking a half pound of soda per week, and yet he has gained in flesh since his imprisonment. It is only in cases where there is no acid on the stomach, that soda proves injurious, and not then perhaps by any caustic effect upon the mucous coat, but rather by overcharging the secretions of the system with an alkaline element. The excess is usually thrown off by the kidneys, otherwise may surcharge the other secretions. In two cases we have known it to irritate the eyes by affecting the lacrymal secretion, causing a smarting and constant tendency to bat them.

---

VULVAR PRURITUS.—M. Marius Key recommends the glycerole of cade as a local application in the treatment of pruritus of the vulva. The formula he employes is one drachm of oil of cade to half an ounce of glycerole of starch. In combination with it he uses tonics, hip-baths and emollient injections, to which laudanum is freely added. He has only tried this treatment in one really rebellious case, but that time with success.—*Gaz. Med. de Paris.*

